# Abnormal Spontaneous Brain Activity in Left-Onset Parkinson Disease: A Resting-State Functional MRI Study

**DOI:** 10.3389/fneur.2020.00727

**Published:** 2020-07-28

**Authors:** Kai Li, Wen Su, Min Chen, Chun-Mei Li, Xin-Xin Ma, Rui Wang, Bao-Hui Lou, Hong Zhao, Hai-Bo Chen, Chuan-Zhu Yan

**Affiliations:** ^1^Department of Neurology, National Center of Gerontology, Beijing Hospital, Beijing, China; ^2^Department of Neurology, Research Institute of Neuromuscular and Neurodegenerative Disease, Qilu Hospital of Shandong University, Jinan, China; ^3^Department of Radiology, National Center of Gerontology, Beijing Hospital, Beijing, China

**Keywords:** Parkinson's disease, resting-state functional MRI (rs-fMRI), amplitude of low frequency fluctuation (ALFF), asymmetry, spontaneous brain activity

## Abstract

**Objective:** Motor asymmetry is characteristic in Parkinson disease (PD). This phenomenon is originated from uneven degeneration of bilateral substantia nigra. However, this asymmetry may not restrict to substantia nigra or striatum. We aimed to determine the effect of asymmetry on spontaneous brain activity across the whole brain.

**Methods:** We consecutively recruited 71 patients with PD, as well as 35 healthy controls, and collected relevant demographic, clinical, and neuropsychological information. The PD patients were divided into two groups according to the side of motor symptom onset. All the participants underwent resting-state functional magnetic resonance imaging, and spontaneous brain activity was assessed using amplitude of low-frequency fluctuation (ALFF). The associations between areas showing significant group differences and various clinical and neuropsychological measures were analyzed.

**Results:** Finally, the data of 30 PD patients with left-onset (LPD), 27 PD patients with right-onset (RPD), and 32 healthy controls were obtained. The three groups had similar age and gender ratios. Our results demonstrated that LPD patients had increased ALFF in the left inferior temporal gyrus and decreased ALFF in bilateral thalamus and cerebellum anterior lobes than the control group. The value of ALFF of the left inferior temporal gyrus was correlated with motor function, and ALFF value of the thalamus was associated with cognition. Comparisons between LPD and RPD patients and between RPD patients and the controls did not yield significant difference.

**Conclusions:** The present study provides new insights into the distinct characteristics of spontaneous brain activity in LPD, which may be associated with motor and cognitive function.

## Introduction

Parkinson disease (PD) is a common neurodegenerative disorder and manifests as motor symptoms including bradykinesia, resting tremor, and rigidity, as well as a series of non-motor symptoms (1). One enigmatic characteristic of PD is the asymmetry of motor symptoms, which presents since disease onset and persists along with disease progression (2, 3). This asymmetry of motor symptoms is unique among various neurodegenerative disorders with parkinsonian syndromes and can serve as a clue for differential diagnosis (4). The mechanism underlying motor symptoms asymmetry is the unequal degeneration of dopaminergic neurons in the midbrain and consistently revealed through autopsy, positron emission tomography, and single-photon emission tomography examinations (5–7).

Furthermore, motor asymmetry also affects non-motor profiles, such as cognitive impairment, psychiatric symptoms, pain, sleep disorders, and olfactory function (8–12). Regarding cognitive function, patients with right-sided PD (RPD) typically have language-related impairments, whereas patients with left-sided PD (LPD) mainly have visuospatial-associated dysfunction (8). The mechanism how lateralization influences non-motor symptoms is still elusive. Unequal impairments of bilateral dopaminergic circuits might partially underlie this phenomenon; more widespread destructions involving multiple brain areas may also account for the impact of lateralization on various non-motor symptoms (8, 13).

A variety of magnetic resonance imaging (MRI) techniques contributes to our improved understanding on structural and functional asymmetric impairment of the brain in PD. A longitudinal study demonstrated that the lateral ventricular volume contralateral to motor symptom onset increased faster than that ipsilateral to motor symptoms onset (14). Magnetic resonance imaging using voxel-based morphometry has revealed that LPD patients had gray matter volume loss primarily in the right hemisphere (15). Diffusion tensor imaging has shown that the substantial nigra contralateral to the side with more severe symptoms had a larger decrease in fractional anisotropy than the opposite side (16). Functional MRI (fMRI) is a valuable tool for the revelation of brain activity and has been used in the exploration of functional substrate of asymmetrical impairment in PD.

Functional MRI can be classified into task-based fMRI and resting-state fMRI (rs-fMRI). The former requires the subject to perform a specific task; the latter needs almost no effort from the patient (17). To date, there have been only few studies employing rs-fMRI in the topic of asymmetry of PD. Tang et al. (18) used whole-brain functional connectivity in mild–moderate PD patients and found that RPD patients had more functional connectivity abnormalities, especially in the brain regions of the left hemisphere belonging to the somatosensory and motor networks, as well as the default mode network. Another study using rs-fMRI analyzed the associations between brain functional connectivity and PD symptoms. They found that Movement Disorder Society (MDS)–Unified PD Rating Scale (UPDRS) part I score was correlated with functional connectivity centered in the inferior orbitofrontal area ipsilateral to the side of more severe motor symptoms, and MDS-UPDRS part III score was correlated with functional connectivity of inferior parietal contralateral to the side of more severe motor symptoms (19). Huang et al. (20) assessed regional homogeneity (ReHo) in the striatum as a measure of brain activity and tested the PD patients' feedback-based associative learning. In their study, LPD patients performed worse than did RPD patients and controls, and this cognitive impairment was associated with ReHo in the right dorsal rostral putamen (20). Amplitude of low-frequency fluctuations (ALFF) is a widely used measure that conveniently reflects regional spontaneous activities of the whole human brain (21). Previous studies on PD using ALFF have indicated that the changes in brain function did not restrict to basal ganglia, but also involved various cerebral areas (22, 23). However, these studies combined LPD and RPD patients as a single group and thus could not reveal differences between spontaneous brain activities of these two subgroups. Therefore, the present study aimed to assess the influence of motor asymmetry on brain activities in PD with the help ALFF.

## Methods

### Participants

From 2012 to 2014, we consecutively enrolled 71 patients with idiopathic PD, and 35 age-, sex-, and general cognitive status–matched controls with no history of neurological or psychiatric disorders. All the patients were diagnosed according to the United Kingdom Parkinson's Disease Society brain bank diagnostic criteria (4).

Demographic and clinical information was collected from all the participants. Side of motor symptom onset was determined by medical history and confirmed by neurological examinations. The patients whose side of onset could not be ascertained would be excluded. Patients with dementia, moderate to severe head tremor, head trauma, deep brain stimulation, alcohol or drug abuse, or with other neurological or psychiatric disorders were excluded. Participants who were left-handed were also excluded from this study.

All the PD patients underwent MRI examination and motor and non-motor function evaluations in a practically defined “off” state, after withdrawing all the antiparkinsonian medications for ~12 h. UPDRS, Hoehn-Yahr staging, Mini-Mental State Examination (MMSE), Hamilton Depression Rating Scale (HAMD), Hamilton Anxiety Rating Scale (HAMA), and Non-Motor Symptoms Questionnaire (NMSQ) were assessed in all the PD patients. The control subjects were evaluated by MMSE.

The study was in accordance with relevant guidelines and regulations and approved by the ethics committee of Beijing Hospital. This study was carried out according to the Declaration of Helsinki. All the subjects gave written informed consent prior to participation.

### MRI Data Acquisition

All the MRI scans were performed on a 3.0-T scanner (Achieva TX; Philips Medical Systems, Best, the Netherlands) at Beijing Hospital. Foam padding and headphones were used to minimize head motion and reduce scanning noise. Participants were asked to lie still, relax, keep their eyes closed, and remain awake through the entire scan. High-resolution T1-weighted images (three-dimensional turbo field echo) were acquired with the following parameters: repetition time (TR) = 7.4 ms, echo time (TE) = 3.0 ms, flip angle = 8°, field of view (FOV) = 240 × 240 mm, matrix size = 256 × 256, voxel dimensions = 0.94 × 0.94 × 1.20 mm, slice thickness = 1.2 mm, 140 slices. Functional images were obtained using axial echo-planar imaging with the following parameters: TR = 3,000 ms, TE = 35 ms, flip angle = 90°, FOV = 240 × 240 mm, matrix size = 64 × 64, voxel dimensions 3.75 × 3.75 × 4.00 mm, slice thickness = 4 mm, slices = 33, time points = 210.

### rs-fMRI Data Preprocessing

Resting-state fMRI data preprocessing and ALFF computation were carried out using RESTplus V 1.2 (24), which is a toolkit based on SPM 12 (http://www.fil.ion.ucl.ac.uk/spm). Generally, the preprocessing pipeline included (1) the first 10 volumes were discarded for participants' acclimatization and signal equilibrium. (2) The remaining 200 time points were corrected for slice-timing. (3) Realignment to account for head motion. Subjects were excluded if their head motion exceeded 2 mm in displacement or 2° in rotation. (4) Functional images were coregistered to the structural T1 images and were normalized to the Montreal Neurological Institute (MNI) template using the coregistered T1 images (by DARTEL) (25). Then they were resliced to a resolution of 3 × 3 × 3 mm^3^. (5) Smoothing using a Gaussian kernel (6-mm full-width-half-maximum, FWHM). (6) Detrending was employed to reduce the systematic shift. (7) Nuisance covariates regression, which regressed out Friston-24 head motion parameters (26), white matter and cerebrospinal fluid signals.

### ALFF Calculations

Amplitude of low-frequency fluctuation was calculated using RESTplus V 1.2, and the algorithms have been published previously (21). Briefly, the time series of all the voxels were converted to the frequency domain using fast Fourier transform. Then the power spectrum was obtained across the frequency of 0.01–0.1 Hz. Afterward, the ALFF value of each voxel was divided by the global mean ALFF value for the standardization across participants. After the ALFF computation, band-pass filtering (0.01 < *f* < 0.1 Hz) was performed to remove the influences of low-frequency drift and high-frequency physiological noise.

### Statistical Analysis

Demographic and clinical variables were analyzed using SPSS (version 23.0; IBM Corp, Armonk, NY, USA). Data are presented as mean ± standard deviation unless stated otherwise. The Kolmogorov–Smirnov test was applied to assess data normality. The one-way analysis of variance or Kruskal–Wallis test was employed to test differences of numerical variables between the LPD, RPD, and control groups. χ^2^ or Fisher exact test was used to compare categorical variables between groups. Differences were considered statistically significant when *p* < 0.05.

Statistical analysis of ALFF was performed using DPABI [Data Processing and Analysis for (Resting-State) Brain Imaging] V4.2 (27). Comparisons between the LPD, RPD, and control groups were conducted using the analysis of covariance model. We used a gray matter mask and set age and gray matter density as covariates to control for the effects of age and structural differences. *Post-hoc* pairwise analyses were corrected by the least significant difference method. Multiple-comparisons corrections were performed based on Gaussian random field theory (voxel-level *P* < 0.001; cluster-level *P* < 0.05; two-tailed) (28, 29). Effect sizes were evaluated using Cohen *f*^2^, which was given by DPABI. We also calculated the statistical power for the clusters having significant between group differences using G^*^Power 3.1.9.7 (30). The ALFF values of clusters showing significant between group differences were extracted, and the relationship between the ALFF values of these positive clusters and clinical and neuropsychological variables was explored via Spearman correlation.

## Results

### Demographic and Clinical Information

Finally, we included 57 patients with PD and 32 controls. Four of the PD patients and two of the controls were excluded because of left-handedness. Five PD patients and one control were excluded because of excessive head motion. One PD patient was excluded because of poor image quality. Four PD patients were excluded because of bilateral motor symptom onset.

Demographic and clinical characteristics are shown in Table 1. There were 30, 27, and 32 subjects in the LPD, RPD, and controls groups, respectively. There was no significant difference in age, sex, or MMSE across the three groups. Disease duration was comparable in PD patients with left- and right-side onset. UPDRS, Hoehn-Yahr staging, HAMD, HAMA, and NMSQ scores were similar between the LPD and RPD groups.

**Table 1:** Demographic and clinical characteristics of the PD patients and controls.

	**LPD**	**RPD**	**Controls**	***p*-value**
No. of subjects	30	27	32	
Age	62.63 ± 8.88	65.85 ± 6.982	62.41 ± 7.07	0.056
Gender (male/female)	14/16	14/13	16/16	0.924
Disease duration	6.80 ± 3.62	6.15 ± 3.59		0.499
Hoehn-Yahr staging	2.13 ± 0.71	2.28 ± 0.67		0.416
UPDRS	49.90 ± 18.82	48.85 ± 12.83		0.809
MMSE	28.50 ± 1.50	27.56 ± 2.28	27.78 ± 2.25	0.203
HAMD	9.07 ± 5.27	9.56 ± 5.09		0.724
HAMA	9.93 ± 5.04	10.52 ± 6.03		0.691
NMSQ	11.07 ± 5.77	11.56 ± 4.86		0.732

### Group Differences in ALFF

Analysis of covariance and the followed *post-hoc* pairwise analyses only identified different brain activities between the LPD and control groups, whereas there was no significant difference between the LPD and RPD groups, or between the RPD and control groups.

**Figure 1:**
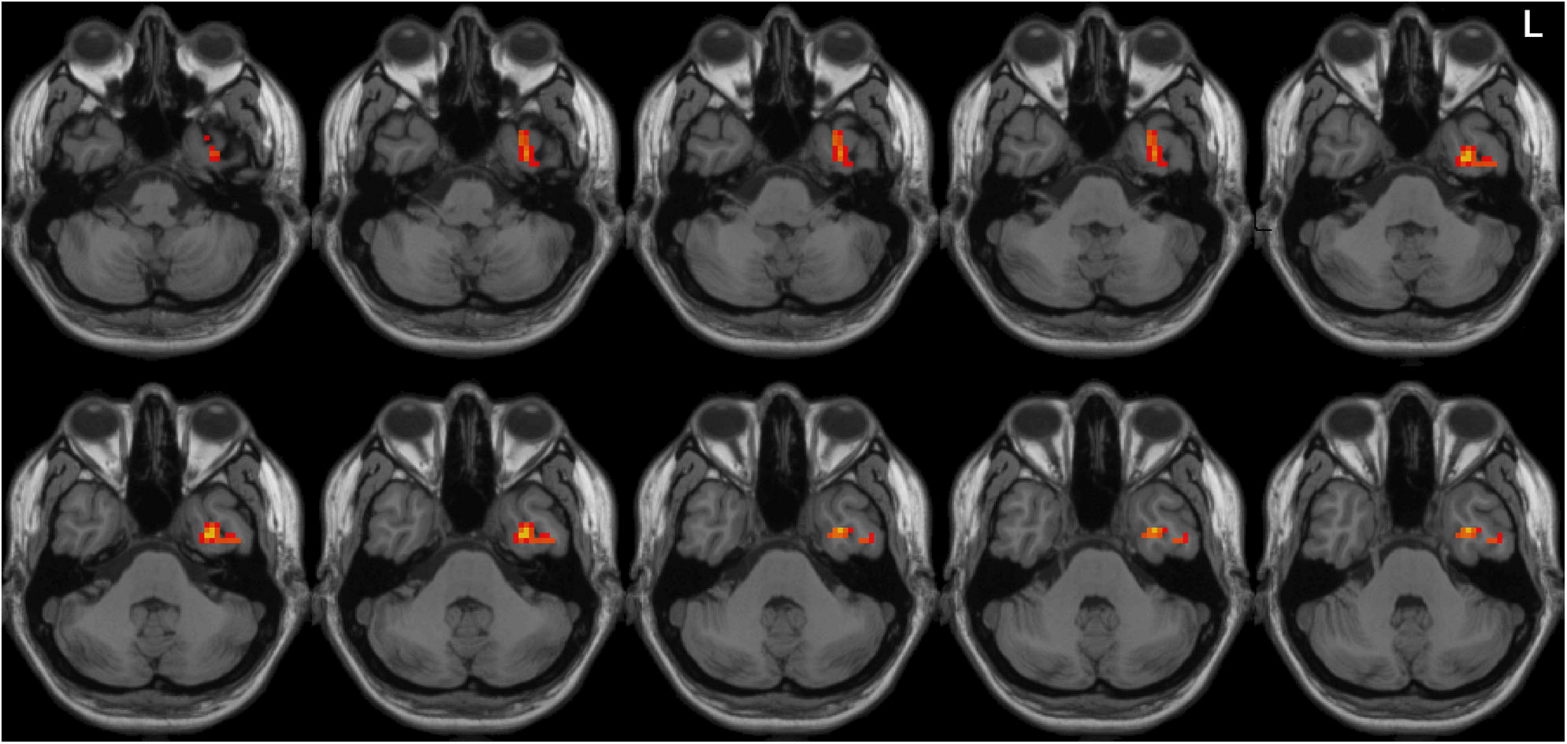
Increased ALFF in the left inferior temporal gyrus in the LPD patients compared with the controls. L indicates the left side.

**Figure 2:**
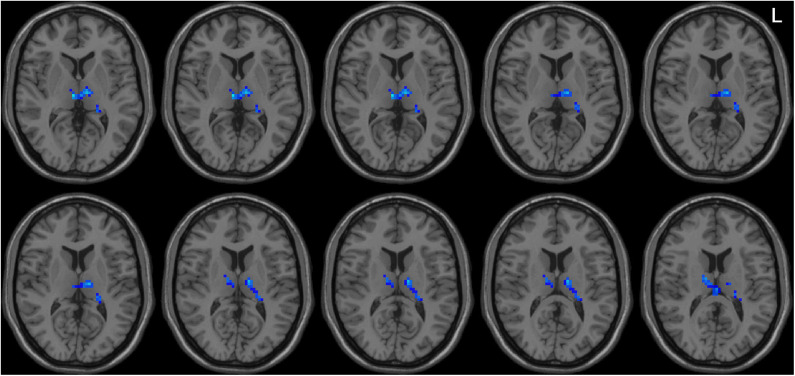
Decreased ALFF in bilateral thalamus in the LPD patients compared with the controls. L indicates the left side.

**Figure 3:**
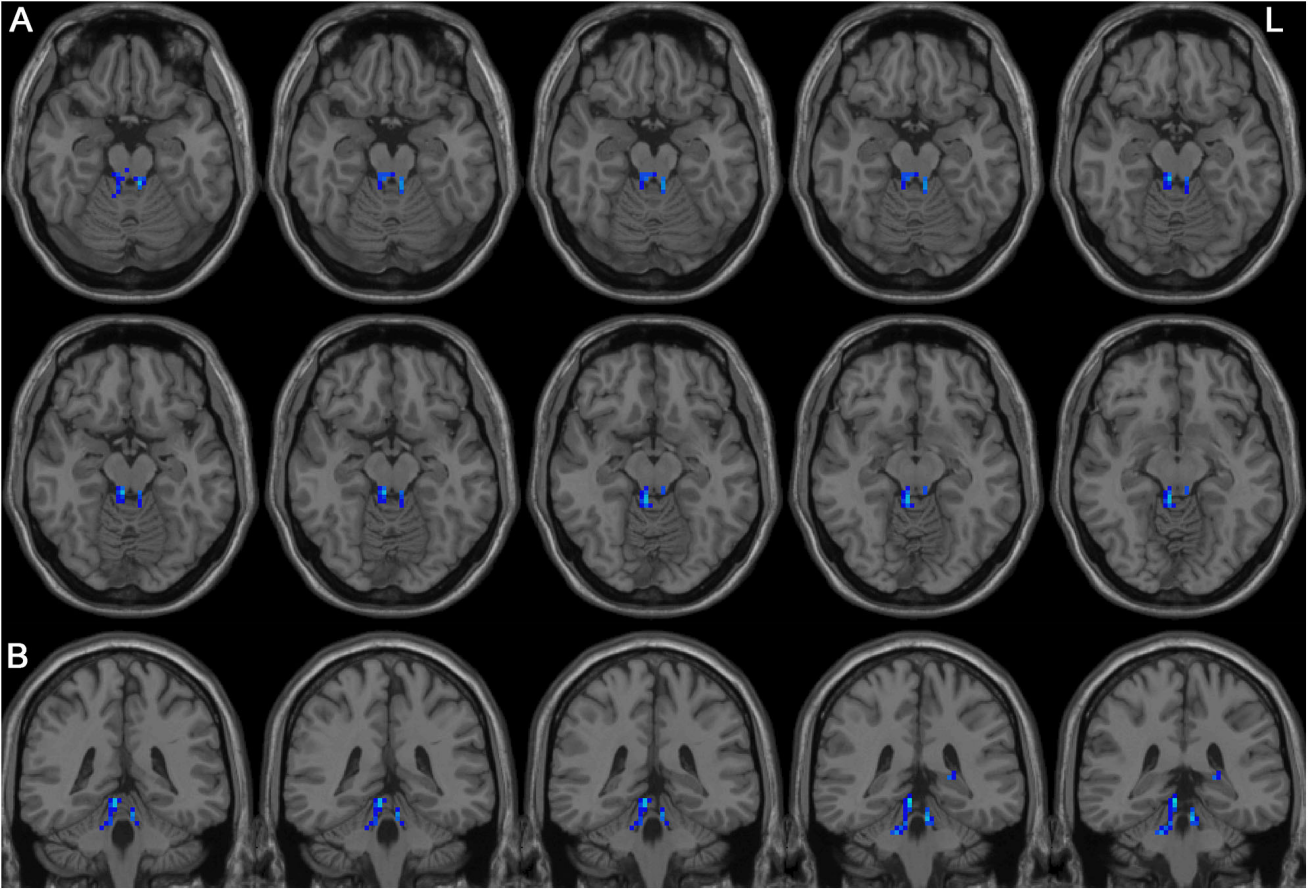
Decreased ALFF in bilateral cerebellum anterior lobes in the LPD patients compared with the controls. **(A)** and **(B)** indicate axial and sagittal views, respectively. L indicates the left side.

Left-sided PD patients exhibited increased ALFF in the left inferior temporal gyrus (Figure 1), as well as deceased ALFF in bilateral thalamus (Figure 2) and cerebellum anterior lobes (Figure 3), compared with the controls. The results are illustrated in Table 2.

**Table 2:** Brain regions with significant differences in ALFF values between LPD and control subjects.

**Brain regions**	**Side**	**Peak MNI coordinates**			**No. of voxels**	***T*-value**	**Effect sizes, Cohen *f*^2^**	**Statistical power**
		***x***	***y***	***z***				
Inferior temporal gyrus	L	−33	−3	−39	59	4.354	0.243	0.9998
Cerebellum anterior lobe	Bilat	6	−36	−9	78	−4.367	0.212	0.9982
Thalamus	Bilat	6	−18	6	155	−4.431	0.207	0.9986

### Correlation Analysis

We performed Spearman correlation analyses between ALFF values of the above three positive clusters and Hoehn-Yahr staging; UPDRS parts I, II, III, and IV; MMSE; HAMD; HAMA; and NMSQ scores in LPD patients. There were three significant correlations: ALFF value of the inferior temporal gyrus was significantly correlated with scores of UPDRS parts II and III (*r* = 0.371, and 0.363; *p* =0.044 and 0.049, respectively); ALFF value of the thalamus was significantly associated with MMSE score (*r* = 0.496, *p* = 0.005). The degree of freedom of the correlation analysis was 28.

## Discussion

This is the first study to investigate the pattern of spontaneous whole-brain activities in PD patients with left and right onset separately. Although directly comparing LPD and RPD patients did not identify significant different regional brain activities, only LPD patients had several areas with abnormal ALFF compared with healthy controls. LPD patients showed increased ALFF in the left inferior temporal lobe, as well as decreased ALFF in bilateral thalamus and cerebellum anterior lobes. Moreover, ALFF value of the left inferior temporal gyrus was associated with scores of activities of daily living and motor parts of UPDRS, and bilateral thalamus ALFF value was associated with cognitive function in the LPD group.

Comparing ALFF values with healthy controls obtained different results for LPD and RPD patients. This difference indicates that the pathological and compensatory mechanisms may be different in LPD and RPD patients. Some studies suggested that LPD and RPD might have different disease severity and speed of progression, and RPD patients had better neural reserve and/or greater neural plasticity than LPD patients (31, 32). In addition, Kim et al. (33) and Lee et al. (15) also found that LPD patients had more areas of atrophy than RPD patients, when the two PD subgroups were compared with control subjects (15, 33). These findings are in concordance with ours that the brains of RPD patients were less impaired than those of LPD patients. It is interesting to follow up the patients, to explore whether widespread aberrant brain activates in the LPD patients would translate to faster progression. The present study underscores the importance of dividing PD patients according to side of onset in future studies, to confirm their distinct brain activity characteristics.

Nevertheless, the direct comparison between LPD and RPD patients did not show significant difference. Moreover, the studies by Kim et al. (33) and Lee et al. (15) also failed to identify areas with significant different cortical thickness or gray matter volume in the direct comparison between LPD and RPD patients, although these two groups showed different patterns of abnormalities compared with the controls (15, 33). We have to be cautious to assert that LPD and RPD have different profiles of brain activity. The mean disease duration was longer than 6 years, and the mean Hoehn-Yahr stage was higher than 2 in both PD groups in the present study. Therefore, most of the PD patients recruited already had bilateral brain impairments. The disparity between the two PD subgroups might be masked by prominent similarities. Although still in controversy, some studies demonstrated that the degree of asymmetry decreased during disease progression (3, 34). Our findings should be considered preliminary results, and further studies including a larger sample and particularly patients with only unilateral symptoms may better reveal the influence of lateralization on cerebral activities in PD.

There were three regions with aberrant ALFF in the comparison between LPD patients and the controls. The Cohen *f* ^2^-values indicated medium effect sizes (0.15 < *f* ^2^ < 0.35) (35), and correlation analysis showed that the altered brain activity in these areas might play a role in motor and non-motor symptoms.

Left-sided PD patients had increased ALFF in the left inferior temporal lobe, and this abnormality was associated motor symptoms and daily activities. Increased ALFF in the left inferior temporal lobe has been reported by several studies (36–39). In the studies by Mi et al. (37), Hu et al. (38), and Chen et al. (39), increased ALFF in this area was associated with motor functions such as posture instability and gait disturbances. It is well-known that the left inferior temporal lobe is critical for semantic integration and visual processing (40, 41). Lahr et al. (42) have demonstrated that PD patients relied on visual input to maintain upright postural control. Integration of visual sensory information is essential for appropriate movement initiation and execution. Inferior temporal lobe dysfunction may influence motor function and daily activities through impaired processing of visual information (37, 42). How does aberrant brain activity of this zone affect motor function in LPD patients needs further investigation.

We found that ALFF of bilateral thalamus was lower in LPD patients than the controls, and ALFF value of thalamus was positively correlated with cognitive function. This positive correlation suggested that the lower the ALFF, the worse the cognitive function. Thus, this abnormality may be a pathological rather than a compensatory change. Thalamus is not only a vital relay of sensory pathways, but also a key component in corticobasal ganglia–thalamic circuits, which modulates motor and cognitive function (43). Decreased thalamic ALFF in PD has been reported in a number of studies (36, 37, 44, 45) and might be associated with depression and gait disturbance (36, 37). Our findings suggest that impaired thalamic activity may be associated with cognitive function in PD. This brain activity change may be mediated through disrupted corticobasal ganglia–thalamic circuit (46), as supported by an rs-fMRI study using functional connectivity (47). Additionally, there is histopathological evidence of thalamic damage and its correlation with cognitive dysfunction in PD (48, 49). Overall, our study confirmed thalamus dysfunction in LPD and suggested its association with cognitive impairment in PD.

The present study revealed lower ALFF in bilateral cerebellum anterior lobes. Cerebellar regulates motor and cognitive function through the cerebellothalamocortical circuit, which has several overlapping structures with the corticobasal ganglia–thalamic loop (40). Mi et al. (37) also revealed decreased ALFF in the cerebellum anterior lobe in PD patients. Wu and Hallett (50) assessed brain activity during automatic movements using fMRI and found increased cerebellar activity of bilateral cerebellum anterior lobes during automatic movements. They suggested that cerebellum might play a compensatory role in automatic movements. We did not find associations between the ALFF value of this area and motor or cognitive function. Maybe a more thorough evaluation especially including automatic movement performance would confirm its role in motor function.

It is noteworthy that there are some limitations of the study. First, the sample size is not very large. We obtained different results in comparisons between the two PD groups and the controls, but directly comparison between LPD and RPD patients did not show any significant difference. This may be due to the small difference between the two PD groups, and a larger sample size may have enough power to detect discrepancies between PD patients with left and right onset. Second, all the patients underwent dopaminergic medications; although we performed rs-fMRI examination in a practically defined off-state, the influence of antiparkinsonism medications cannot be completely ruled out. However, this practically defined off-state is quite commonly used in rs-fMRI studies in PD and would facilitate comparisons with other studies in this topic.

In conclusion, the present study showed that LPD had abnormalities in spontaneous brain activities in the left temporal lobe, bilateral thalamus, and cerebellum anterior lobes, and some of these changes were related to motor and cognitive function. However, RPD and healthy controls did not have significant difference in brain activities measured by ALFF. This finding indicates that LPD and RPD might have different neural mechanisms during neurodegeneration. Furthermore, this study highlights the necessity of dividing PD patients according to side of onset, to detect the characteristic pathophysiology of these two subgroups of PD.

## Data Availability Statement

The datasets generated for this study are available on request to the corresponding author.

## Ethics Statement

The studies involving human participants were reviewed and approved by the Ethics Committee of Beijing Hospital. The patients/participants provided their written informed consent to participate in this study.

## Author Contributions

WS, H-BC, and C-ZY conceived and designed the experiments. KL analyzed the fMRI data. MC, C-ML, RW, and B-HL were responsible for the fMRI scans and helped fMRI data analyses. WS and H-BC recruited the subjects. X-XM, HZ, and KL collected the demographic, clinical, and neuropsychological information of the subjects. KL and WS wrote the manuscript. All the authors have read and approved the final manuscript.

## Conflict of Interest

The authors declare that the research was conducted in the absence of any commercial or financial relationships that could be construed as a potential conflict of interest.
